# Cerebrospinal fluid alpha-internexin concentrations measured in patients with Guillain–Barré syndrome: a possible prognostic biomarker for disability at 12 months

**DOI:** 10.1007/s00415-026-13789-y

**Published:** 2026-04-07

**Authors:** Nina Gleisner, Francisco Meda, Magnus Johnsson, Igal Rosenstein, Ellen Alpsten, Lenka Novakova, Jan Lycke, Henrik Zetterberg, Hlin Kvartsberg, Brynhildur Hafsteinsdottir, Markus Axelsson

**Affiliations:** 1 Department of Clinical Neuroscience, Institute of Neuroscience and Physiology, The Sahlgrenska Academy, University of Gothenburg, Gothenburg, Sweden; 2 Department of Neurology, Sahlgrenska University Hospital, Region Västra Götaland, Gothenburg, Sweden; 3 Department of Psychiatry and Neurochemistry, Institute of Neuroscience and Physiology, The Sahlgrenska Academy, University of Gothenburg, Mölndal, Sweden; 4 Clinical Neurochemistry Laboratory, Sahlgrenska University Hospital, Mölndal, Sweden; 5 Department of Neurodegenerative Disease, UCL Institute of Neurology, Queen Square, London, UK; 6 UK Dementia Research Institute at UCL, London, UK; 7 Hong Kong Center for Neurodegenerative Diseases, Clear Water Bay, Hong Kong, China; 8 Wisconsin Alzheimer’s Disease Research Center, School of Medicine and Public Health, University of Wisconsin, University of Wisconsin-Madison, Madison, WI USA; 9 Neurodegenerative Disorder Research Center, Division of Life Sciences and Medicine, and Department of Neurology, Institute on Aging and Brain Disorders, University of Science and Technology of China and First Affiliated Hospital of USTC, Hefei, China; 10 Department of Pathology and Laboratory Medicine, University of Wisconsin School of Medicine and Public Health, Madison, WI USA; 11 Centre for Brain Research, Indian Institute of Science, Bangalore, India

**Keywords:** Guillain–Barré syndrome, Biomarkers, Alpha-internexin, Prognosis

## Abstract

**Background:**

There is an unmet need for predictive biomarkers in Guillain–Barré syndrome (GBS). We aimed to determine whether cerebrospinal fluid (CSF) alpha-internexin (AINX) concentrations are associated with disease severity and outcome in GBS.

**Methods:**

This retrospective cohort included 100 GBS patients. AINX concentrations were measured at diagnosis using a sensitive Simoa assay. Disease severity at nadir was assessed with the GBS Disability Scale (GBSDS) and the need for mechanical ventilation. Poor outcome was defined as GBSDS > 2 at 12 months. Logistic regression with log-transformed AINX assessed associations with severe disease at nadir (GBSDS > 2), need for mechanical ventilation, and poor outcome at 12 months (GBSDS > 2). ROC curve analysis assessed the ability of AINX to predict poor outcome and compared its performance with previously reported biomarkers re-analyzed in this cohort.

**Results:**

AINX concentrations at diagnosis were higher in patients with poor outcome and remained associated with poor outcome after adjustment for age (OR 12.48, 95% CI 1.20–129.1, *p* = 0.034). This association remained significant after additional adjustment for axonal subtypes (OR 26.38, 95% CI 1.12–619.77, *p* = 0.042). ROC analysis demonstrated good discriminative performance (AUC of 0.79, 95% CI 0.61–0.97, *p* = 0.002). The optimal cutoff of 2.9 ng/L yielded 50% sensitivity and 95.7% specificity. AINX concentrations were not associated with disease severity at nadir or with need for mechanical ventilation.

**Conclusions:**

Elevated CSF AINX at diagnosis was associated with poor long-term outcome in GBS, consistent with a possible contribution of CNS-related injury to recovery.

## Introduction

Guillain–Barré syndrome (GBS) is considered a monophasic neurological disease characterized by acute inflammation of peripheral nerves and nerve roots, typically reaching disease nadir within four weeks [1]. The clinical presentation is heterogeneous and several subgroups have been described [2]. Disease severity and outcomes vary between individuals, and there is an unmet need for reliable predictive biomarkers. Neurophysiological axonal subtypes have previously been linked to less favorable long-term outcomes [3]. Although GBS is generally considered a disease of the peripheral nervous system (PNS), involvement of the central nervous system (CNS) has been recognized, particularly in variants such as Miller Fisher syndrome (MFS) [4], and in cases with hyperreflexia where an involvement of spinal interneurons or the corticospinal tract has been suggested [5].

Neurofilaments (Nf) are intermediate filaments which function to stabilize the neuroaxonal cytoskeleton. Seven isoforms have been identified in humans, each encoded by different genes on separate chromosomes. The major isoforms, neurofilament light chain (NfL), two variants of neurofilament medium chain (NfM), and two variants of neurofilament heavy chain (NfH), are expressed in axons in both the CNS and PNS. In contrast, alpha-internexin has been described to be restricted to the CNS [6], whereas peripherin is almost exclusively expressed in peripheral nerve axons [7].

AINX has been detected in neuronal intracytoplasmic intermediate filament inclusions in several neurodegenerative disorders, most prominently in neuronal intermediate filament inclusion disease (NIFID), and more rarely in other conditions such as Alzheimer’s disease and Lewy body dementia [8]. Expression of AINX has also been detected in medulloblastomas [9], neuroblastomas [10], and certain types of gastroenteropancreatic neuroendocrine neoplasms (GEP-NENs) [11], likely reflecting shared neuroectodermal features. More recently, Johnsson et al. reported significantly elevated AINX concentrations in patients with multiple sclerosis (MS), a well-established CNS disorder, compared with healthy controls [12]. To our knowledge, an association between AINX and GBS has not previously been described.

We previously demonstrated that elevated concentrations of the CNS biomarker brain-derived tau (BD-tau) are associated with long-term outcome in GBS [13]. These findings raise the question of whether CNS involvement may influence recovery and disease severity in GBS. The aim of the present study was to investigate whether CSF AINX concentrations at diagnosis are associated with 12-month outcome in patients with GBS.

## Methods

### Study design and patients

The study was a retrospective cohort study. The study population consisted of 100 patients diagnosed with GBS in the Västra Götaland region of Sweden between 2011 and 2021, identified through the ICD diagnostic code for GBS (G61.0) in local and national patient registers. This cohort has previously been described in part, including analyses of serum and CSF neurofilament light chain (sNfL, CSF NfL), serum and CSF brain-derived tau (sBD-tau, CSF BD-tau), and Qalb [13]. The present study includes additional cases and investigates AINX, which has not previously been examined in this cohort. Through cross-referencing with a clinical database at the regional microbiology laboratory of Sahlgrenska University Hospital, cases with stored samples in the laboratory biobank were identified. Medical records were reviewed to confirm GBS diagnosis according to the Brighton diagnostic criteria [14]. Patients with coexisting neurological diseases were excluded.

Functional status in patients with GBS at diagnosis, at disease nadir, and at 12 months was retrospectively assessed through medical record review using the GBS Disability Scale (GBSDS) [15]. Disease severity at nadir was defined as functional impairment (GBSDS > 2) and/or the requirement for mechanical ventilation. Poor outcome was defined as inability to walk without aid (GBSDS > 2) at 12 months, in line with classifications used in prior studies [13, 16, 17]. Neurophysiological subtypes were classified as AIDP, AMAN/AMSAN, Miller Fisher variant, normal, or equivocal based on diagnostic nerve conduction studies interpreted by board-certified neurophysiologists. Clinical classification was performed according to the Wakerley criteria [18]. For patients included from the previously described cohort [13], the subtype classifications were retained, while new cases were classified by using the same criteria (classic, paraparetic, Miller Fisher syndrome, and the pharyngeal–cervical–brachial variant). Magnetic resonance imaging (MRI) of the lumbosacral spine was performed as part of routine clinical evaluation in a subset of patients. MRI reports were retrospectively reviewed for the presence of hyperemic lumbosacral nerve root swelling, as documented by the reporting radiologist. Patients with MFS were excluded from MRI-based analyses due to their distinct clinical phenotype and limited peripheral nerve root involvement. The presence of fatigue at 12 months was assessed unsystematically by the treating physicians based on self-reported symptoms from the patients without use of a standardized instrument and retrospectively collected from medical records.

### Sample collection and biomarker analysis

CSF samples were collected at diagnosis as part of the routine diagnostic workup and stored at − 20 °C until analysis. AINX measurements were performed in a blinded and randomized manner using a newly developed assay at the University of Gothenburg. This assay uses the ultra-sensitive single molecule array platform (Simoa) platform and in-house produced antibodies, as described previously [19]. Concentrations below the lower limit of quantification (LLOQ; 0.137 ng/L; *n* = 15) were imputed as LLOQ/2 (0.069 ng/L) for analyses.

Measurements of Qalb, sNfL, CSF NfL, sBD-tau, and CSF BD-tau in this cohort have been reported previously [13]. In brief, NfL concentrations were measured using the Simoa NF-light Advantage Kit (Quanterix), albumin by immunonephelometry, and BD-tau using the previously described Simoa method [19]. In the present study, analyses were restricted to patients with available AINX measurements, resulting in partially overlapping but not identical study populations. Published biomarkers were re-analyzed to assess correlations and comparative performance with AINX. Sample sizes for each analysis are provided in the corresponding figure legends.

### Statistical analysis

Statistical analyses were performed using IBM SPSS (version 29.0.2.0) and GraphPad Prism (version 10.6.0). As AINX concentrations were non-normally distributed, data are presented as median and interquartile range (IQR), and log-transformed concentrations were used in parametric analyses. Associations between biomarkers and continuous variables were assessed using Spearman’s rank correlation coefficient (ρ) with 95% confidence intervals (CI). Correlation analyses included associations between AINX and age, AINX and storage time, and between CSF BD-tau and storage time. Inter-biomarker correlations were assessed among AINX, Qalb, NfL (serum and CSF), and BD-tau (serum and CSF). Given the previously reported influence of storage time on CSF BD-tau [13], CSF BD-tau concentrations were adjusted for storage time using linear regression prior to subsequent analyses.

Differences in AINX concentrations across age groups were assessed using one-way ANOVA on log-transformed concentrations. Associations between AINX concentrations and categorical clinical variables, including MRI findings, were evaluated using ANCOVA with age at diagnosis included as a covariate.

Logistic regression analyses were performed using log-transformed AINX concentrations as the main predictor. Separate models were constructed for severe disease at nadir, need for mechanical ventilation, and poor outcome at 12 months. Models were adjusted for age at diagnosis. Odds ratios correspond to a one-unit increase in log-transformed AINX concentrations. To evaluate the independent prognostic value of AINX for poor outcome at 12 months, an additional multivariable model including log-transformed AINX concentrations, age at diagnosis, and neurophysiological axonal subtype (AMAN/AMSAN vs other subtypes) was constructed. Age and axonal subtype were included based on their established association with prognosis [3, 20].

Receiver operating characteristic (ROC) curve analyses were performed using log-transformed biomarker concentrations (AINX, sNfL, storage-time-adjusted CSF BD-tau residuals, and Qalb) to evaluate discriminative ability of poor outcome at 12 months. Discriminative ability was assessed by the area under the curve (AUC). Optimal cutoffs were determined using Youden’s index. For biomarkers analyzed on the log scale, cutoffs were back transformed to the original scale for clinical interpretability (ng/L for AINX and sNfL). For storage-time-adjusted CSF BD-tau, residual values were used for ROC analyses. Corresponding sensitivity and specificity were reported. A *p* value < 0.05 was considered statistically significant.

### Standard protocol approvals, registrations, and patient consents

Informed consent for this study was not required since the data are anonymized and reported at group level. According to local clinical practice, samples collected for microbiological testing are stored in a biobank for up to 10 years and may be used for future research. Patients can decline storage of their samples in the biobank at the time of sampling. The study was conducted in accordance with the Code of Ethics of the World Medical Association (Declaration of Helsinki) [21]. Ethical approval was obtained from the Swedish Ethical Review Authority (Dnr 2020–02558 and 2021–04229).

## Results

A total of 100 patients with GBS were included in the study. Availability of previously published biomarker data differed across analyses due to variations in sample availability. The mean age was 52 years (range 3–87). Three patients were younger than 18 years (aged 3, 15, and 17). MRI was performed in 53 cases (excluding MFS cases), showing hyperemic nerve root swelling in 7 and no swelling in 46. Log-transformed AINX concentrations were not associated with MRI signs of hyperemic nerve root swelling after adjustment for age (*p* = 0.286).

**Fig. 1:**
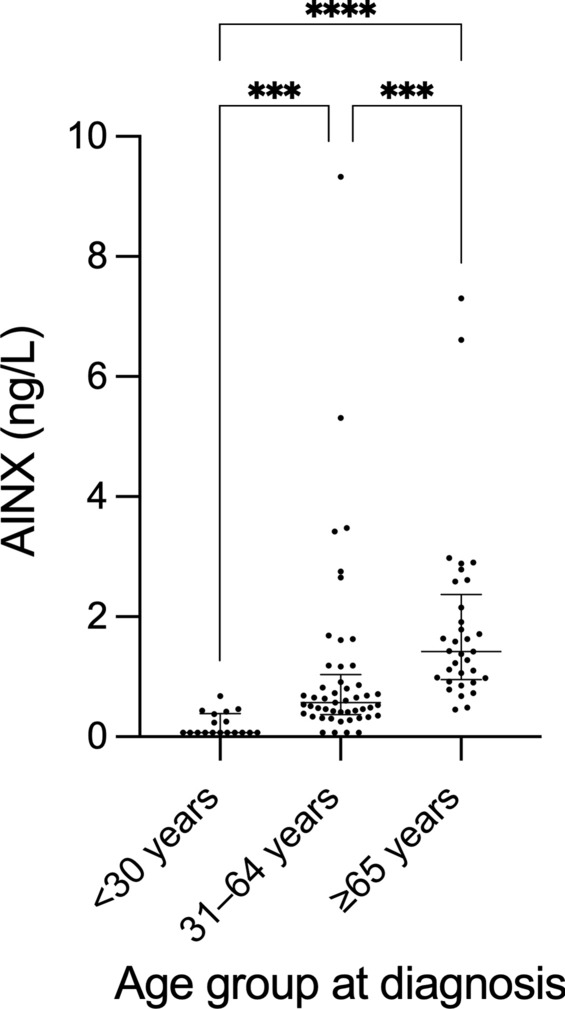
Age group at diagnosis and AINX concentrations. Distribution of AINX concentrations across age groups at diagnosis; dots represent individual patients; horizontal lines indicate medians with interquartile ranges; ***p < 0.001, ****p < 0.0001. *AINX* alpha-internexin

There was no significant correlation between storage time and AINX concentrations (Spearman’s *ρ* = 0.059, *p* = 0.561). In contrast, CSF BD-tau showed a significant association with storage time and was adjusted accordingly in subsequent analyses. The median time from clinical onset to sampling was 6 days (IQR 3–14). Sampling was performed prior to treatment initiation in 45 patients, on the same day in 38 patients, and after treatment initiation in 13 patients. In four patients, the timing could not be determined. AINX concentrations correlated with age as a continuous variable (Spearman´s *ρ* = 0.773, *p* < 0.001). Consistently, when patients were stratified into age groups (< 30 years, 31–64 years, and ≥ 65 years), AINX concentrations increased significantly across groups, with higher concentrations in older age categories (Fig. 1, *p* = 0.006). Aside from age, AINX concentrations did not differ significantly across other demographic or clinical characteristics (Table 1).

**Table 1:** Demographic and clinical characteristics with association to AINX concentrations

Clinical characteristics	*N*	AINX (ng/L), median (IQR)	*p* value
Sex			
Male	67	0.73 (0.39–1.64)	
Female	33	0.57 (0.31–1.40)	*p* = 0.997
Brighton criteria level			
1	45	0.73 (0.42–1.60)	
2	38	0.56 (0.33–1.39)	
3	17	0.65 (0.19–1.85)	*p* = 0.913
Treatment			
IVIG	91	0.65 (0.33–1.42)	
Plasmapheresis	6	0.98 (0.86–4.32)	
None	3	0.51 (*–)	*p* = 0.258
Age group			
< 30 years	18	0.07 (0.07–0.39)	
31–64 years	49	0.57 (0.37–1.04)	
≥ 65 years	33	1.42 (0.95–2.37)	*p* = 0.006
GBSDS at diagnosis			
1	27	0.48 (0.25–0.82)	
2	40	0.71 (0.43–1.62)	
3	20	0.98 (0.50–2.50)	
4	10	0.87 (0.32–1.91)	*p* = 0.378
GBSDS at disease nadir			
1	10	0.46 (0.28–1.24)	
2	24	0.54 (0.25–0.89)	
3	17	0.64 (0.44–1.01)	
4	30	1.09 (0.62–2.60)	
5	16	0.54 (0.29–1.75)	
6	1	1.63 (*–)	*p* = 0.969
GBSDS at 12 months			
0	42	0.44 (0.07–0.87)	
1	27	0.80 (0.46–1.12)	
2	10	1.04 (0.32–1.92)	
3	3	0.68 (*–)	
4	4	4.79 (1.11–8.65)	
5	1	5.31(*–)	
6	4	2.21 (0.92–2.87)	*p* = 0.082
Clinical subtype			
Classic	83	0.68 (0.34–1.43)	
Paraparetic	8	0.79 (0.28–1.14)	
PCB	2	0.99 (*–)	
MFS	7	0.85 (0.44–1.79)	*p* = 0.730
Neurophysiologic subtype			
AIDP	62	0.70 (0.34–1.42)	
AMAN/AMSAN	8	1.31 (0.65–5.68)	
MFS	6	0.67 (0.44–1.90)	
Normal	13	0.45 (0.19–0.85)	
Equivocal	4	1.06 (0.36–2.97)	*p* = 0.261
Preceding infection			
Respiratory	30	0.58 (0.31–1.12)	
Gastrointestinal	19	0.65 (0.34–2.89)	*p* = 0.590
Hyperemic nerve roots on MRI			
No	46	0.65 (0.38–1.31)	
Yes	7	0.07 (0.07–2.65)	*p* = 0.286
Fatigue at 12 months			
No	88	0.69 (0.35–1.61)	
Yes	7	0.46 (0.30–0.60)	*p* = 0.386

*GBSDS* Guillain–Barré syndrome disability scale, *PCB* pharyngeal–cervical–brachial variant, *MFS* Miller Fisher syndrome, *AIDP* acute inflammatory demyelinating poly neuropathy, *AMAN/AMSAN* acute motor/sensory axonal neuropathy

*-IQR was not calculated with *n* ≤ 3

### AINX concentrations and outcome

**Fig. 2:**
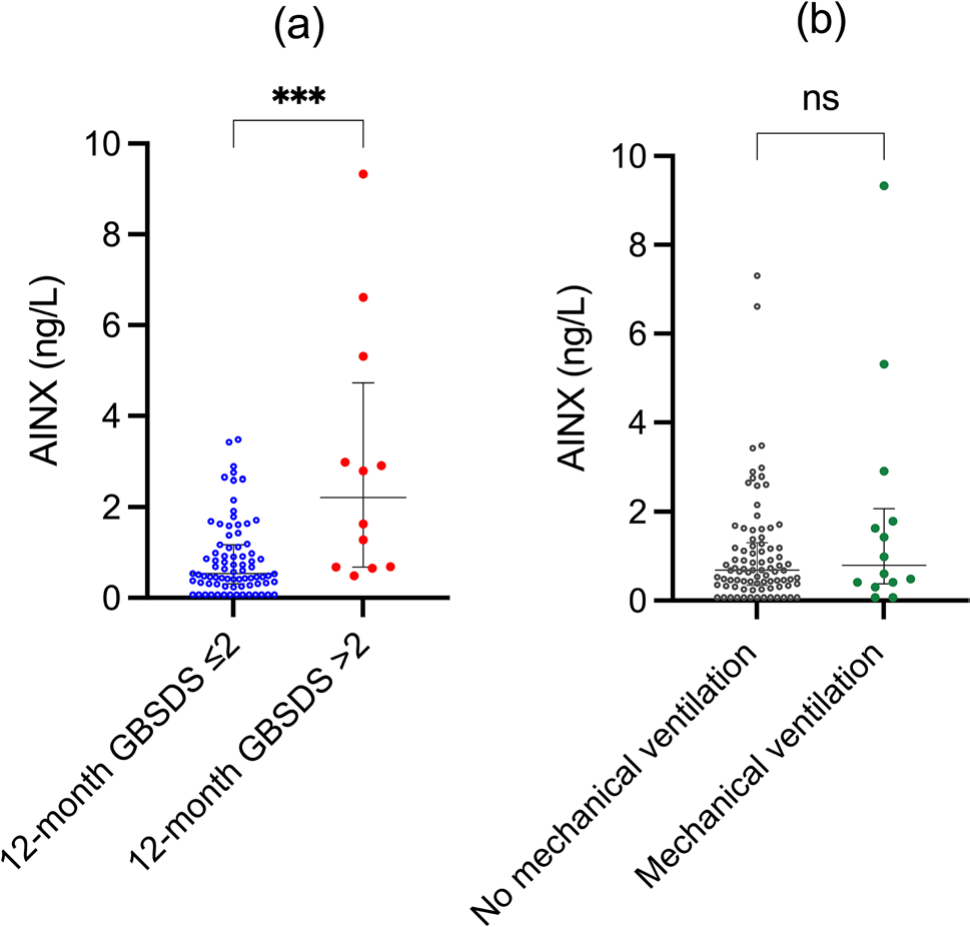
AINX concentrations at diagnosis in relation to clinical outcomes in GBS. **a** AINX concentrations at diagnosis stratified by functional outcome at 12 months, defined as GBS Disability Scale (GBSDS) ≤ 2 versus > 2 (GBSDS ≤ 2, *n* = 79; GBSDS > 2, *n* = 12). **b** AINX concentrations at diagnosis stratified by requirement for mechanical ventilation during the acute disease phase (no mechanical ventilation, *n* = 86; mechanical ventilation, *n* = 14). Dots represent individual patients; horizontal lines indicate median (IQR). *ns* not significant; ***p < 0.001. *GBSDS* Guillain–Barré disability scale

AINX concentrations at diagnosis were significantly higher in patients with poor outcome at 12 months compared with those with a more favorable outcome (median 2.21 [IQR 0.68–4.73] vs 0.54 [0.31–1.17] ng/L). In logistic regression analyses adjusted for age, a one-unit increase in log-transformed AINX concentrations at diagnosis was associated with a 12.5-fold increase in the odds of poor outcome at 12 months (OR 12.5, 95% CI 1.21–129.1, *p* = 0.034) (Fig. 2a). In the multivariable model additionally including axonal subtype, AINX remained independently associated with poor outcome (Table 2).

**Table 2:** Logistic regression analyses of predictors of poor outcome at 12 months

Variable	OR (95% CI)	*p* value
Unadjusted analyses—GBSDS > 2 at 12 months		
CSF AINX^†^	22.39 (3.34–149.97)	*p* = 0.001
Age at diagnosis	1.10 (1.03–1.17)	*p* = 0.003
Axonal subtype	18.06 (3.44–94.75)	*p* < 0.001
Multivariable model—GBSDS > 2 at 12 months		
CSF AINX^†^	26.38 (1.12–619.77)	*p* = 0.042
Age at diagnosis	1.14 (1.03–1.28)	*p* = 0.014
Axonal subtype	25.94 (2.39–281.42)	*p* = 0.007

^†^Calculations performed on logarithmically transformed CSF AINX. Axonal subtype coded as AMAN/AMSAN vs other subtypes. The multivariable logistic regression model was significant (likelihood ratio test: *χ*^2^ (3) = 26.39, *p* < 0.001), with model fit expressed as Nagelkerke *R*^2^ = 0.52. Sample size varied between analyses due to missing data (AINX *n* = 91; age *n* = 88; axonal subtype *n* = 79; multivariable model *n* = 77)

No significant difference in AINX concentrations at diagnosis was observed between patients with severe disease at nadir and those without (median 0.80 [IQR 0.43–1.64] vs 0.50 [0.25–0.96] ng/L, *p* = 0.860), nor between patients who required mechanical ventilation and those who did not (0.79 [0.38–2.07] vs 0.69 [0.35–1.31] ng/L, *p* = 0.547) (Fig. 2b).

In unadjusted logistic regression analyses, higher log-transformed AINX concentrations, older age at diagnosis, and axonal subtype were each associated with poor outcome at 12 months. These associations remained significant in the multivariable logistic regression model after mutual adjustment for age and axonal subtype (Table 2).

### Discriminative performance assessed by ROC curves

**Fig. 3:**
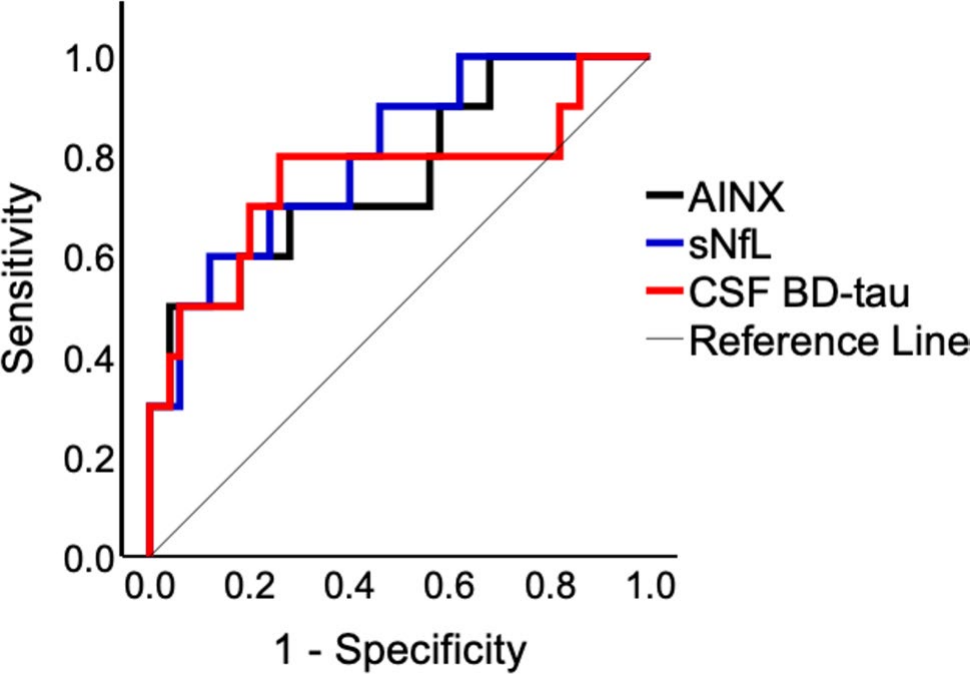
ROC curve analyses based on log-transformed biomarker concentrations (AINX, sNfL, and CSF BD-tau) for prediction of inability to walk independently (GBSDS > 2) at 12 months. Sample sizes were AINX (*n* = 100), sNfL (*n* = 92), and storage-time-adjusted CSF BD-tau (*n* = 70). *AINX* alpha-internexin, *sNfL* neurofilament light chain in serum, *CSF BD-tau* brain-derived tau in cerebrospinal fluid

ROC curve analysis was performed using log-transformed biomarker concentrations to evaluate the discriminative ability of each biomarker for poor outcome at 12 months. AINX concentrations at diagnosis showed good discriminative performance with an AUC of 0.79 (95% CI 0.61–0.97, *p* = 0.002). The optimal cutoff derived from Youden´s index (2.9 ng/L) yielded a sensitivity of 50% and a specificity of 95.7% for predicting poor outcome at 12 months. Discriminative performance was comparable to sNfL (AUC 0.77 95% CI 0.59–0.95, *p* = 0.003) and storage-time-adjusted CSF BD-tau (AUC 0.80 95% CI 0.61–0.99, *p* = 0.002), whereas Qalb showed poor and non-significant discrimination (AUC 0.58 95% CI 0.32–0.84, *p* = 0.557). ROC curves for AINX, sNfL, and storage-time-adjusted CSF BD-tau are shown in Fig. 3, and detailed ROC metrics are provided in Table 3.

**Table 3:** Youden’s index-derived optimal cutoff values with corresponding sensitivities and specificities for AINX, sNfL, adjusted CSF BD-tau (residual), and Qalb in ROC analyses predicting poor functional outcome at 12 months

Biomarkers	AUC (95% CI)	*p* value	Youden´s index	Cutoff	Sensitivity	Specificity
AINX	0.79 (0.61–0.97)	0.002	0.457	2.9	50%	95.7%
sNfL	0.77 (0.59–0.95)	0.003	0.457	600	50%	95.7%
Adjusted CSF BD-tau (residual)	0.80 (0.61–0.99)	0.002	0.592	0.18	88%	72%
Qalb	0.58 (0.32–0.84)	0.557	0.321	15	63%	70%

Adjusted CSF BD-tau represents residual values from linear regression adjusting for storage time

*AINX* alpha-internexin (ng/L), *sNfL* neurofilament light chain in serum (ng/L), *CSF BD-tau* brain-derived tau in cerebrospinal fluid, *Qalb* albumin quotient

### Correlation with other biomarkers

**Fig. 4:**
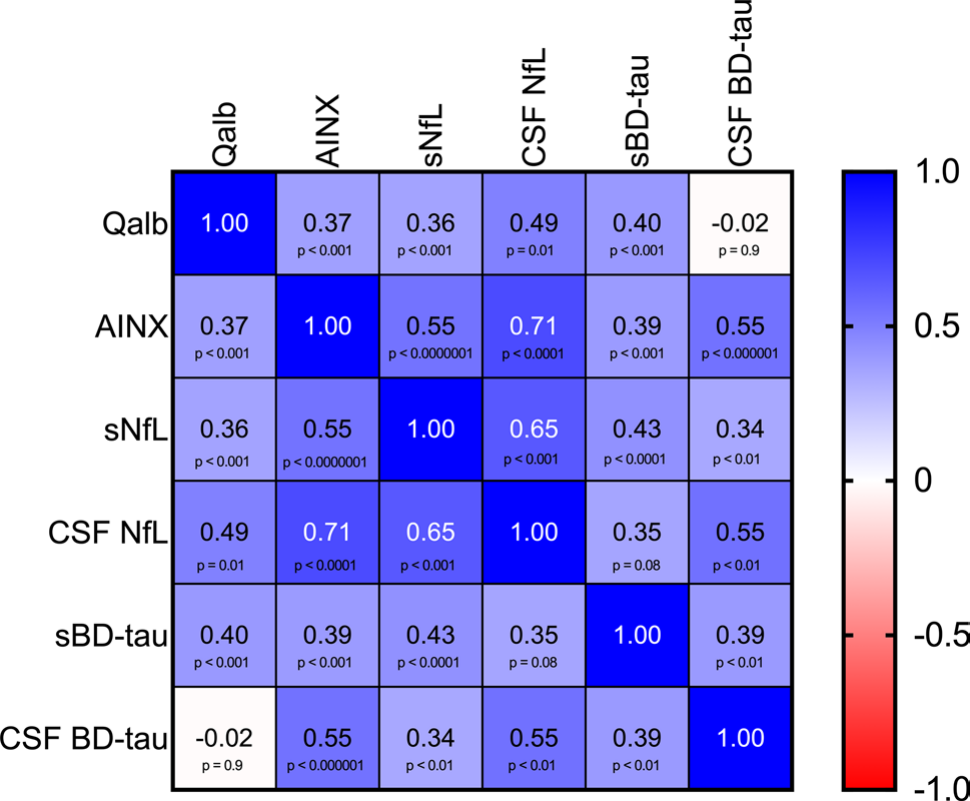
Correlations between biomarkers. Spearman correlation coefficients (ρ) with corresponding *p* values for Qalb, AINX, sNfL, CSF NfL, sBD-tau, and CSF BD-tau. The previously published biomarkers were re-analyzed in patients with available AINX measurements [13]. Sample sizes were as follows: AINX (*n* = 100), Qalb (*n* = 88), sNfL (*n* = 92), CSF NfL (*n* = 28), sBD-tau (*n* = 89), and CSF BD-tau (*n* = 70). *Qalb* albumin quotient, *AINX* alpha-internexin, *sNfL* neurofilament light chain in serum, *CSF NfL* neurofilament light chain in cerebrospinal fluid, *sBD-tau* serum BD-tau, *CSF BD-tau* brain-derived tau in cerebrospinal fluid

Spearman’s rank correlation analysis was performed for Qalb, AINX, sNfL in and CSF, as well as for BD-tau in serum and CSF. CSF BD-tau values were adjusted for storage time using linear regression. Results showed statistically significant positive correlations between AINX concentrations and all other included biomarkers. Correlations between storage-time-adjusted CSF BD-tau and Qalb were negative, however, not significant. Correlations between CSF NfL and sBD-tau were also not significant. All remaining correlations were positive and statistically significant. Spearman´s rho coefficients with corresponding *p* values are shown in Fig. 4.

## Discussion

Our study demonstrated that elevated AINX concentrations at diagnosis were independently associated with poor long-term outcome in GBS. AINX concentrations increased with age, consistent with previous observations for NfL [22]. No correlation with storage time was observed, supporting the stability of AINX as a biomarker. In multivariable analyses, AINX remained independently associated with poor outcome, with a magnitude of association comparable to that observed for sNfL and CSF BD-tau [13].

We did not observe any significant association between AINX concentrations at diagnosis and disease severity at nadir, nor were concentrations significantly elevated in patients requiring mechanical ventilation. The absence of an association with the need for mechanical ventilation may suggest that respiratory impairment in GBS is primarily related to peripheral phrenic nerve involvement rather than central mechanisms, consistent with earlier findings linking phrenic nerve conduction abnormalities to respiratory dysfunction [23].

AINX concentrations were not associated with MRI signs of nerve root hyperemia or with functional status at diagnosis. These findings suggest that AINX concentrations may not simply reflect acute disease severity at presentation. Instead, given the reported CNS enrichment of alpha-internexin, elevated AINX concentrations may reflect proximal or central neuroaxonal involvement that could influence long-term recovery.

The initial lesion in GBS is thought to occur within the proximal nerve roots, where inflammatory endoneurial edema may induce ischemia and subsequent axonal or myelin injury. In axonal forms (AMAN/AMSAN), this process can lead to Wallerian-like degeneration that may propagate both distally along peripheral nerves and proximally toward the intrathecal roots and adjacent spinal cord tracts [24]. Such mechanisms could provide a plausible explanation for early elevations of CNS-derived biomarkers, including AINX and CSF BD-tau; the latter has previously been linked to poor outcome in GBS [13]. Although retrograde degeneration has been described in axonal cases, these represent only a small proportion of our cohort. In other variants, secondary involvement of corticospinal pathways cannot be entirely excluded, as suggested by the observed biomarker elevations. Disruption of the blood-CSF barrier (BCSFB) with a normal cell count is a hallmark of GBS and has previously been linked to disease severity, potentially affecting biomarker distribution [25]. The precise transition zone between the nervous systems remains uncertain. Electron microscopy studies of rat dorsal rootlets have shown that the CNS-PNS boundary varies in length and therefore cannot be clearly defined [26].

AINX significantly correlated with other biomarkers previously identified as predictors of outcome in GBS [13, 27–29]. The associations were strongest with CSF biomarkers, particularly with NfL (*ρ* = 0.71), and more moderate with sNfL and BD-tau. Although sNfL, CSF BD-tau, and AINX demonstrated similar prognostic accuracy (AUC 0.77–0.80), the moderate correlation between CSF BD-tau and AINX (*ρ* = 0.55) suggests that they may reflect related but partly distinct aspects of neuroaxonal injury. The strong correlation between AINX and CSF NfL, together with only a modest correlation with Qalb (*ρ* = 0.37), indicates that AINX concentrations are unlikely to be primarily driven by BCSFB dysfunction and may instead reflect intrathecal release. Although AINX has been described as predominantly expressed in the CNS, definitive evidence for strict CNS specificity in humans remains limited [6, 7, 30]. In this context, our findings raise the possibility that elevated AINX concentrations at diagnosis may reflect CNS-related axonal involvement in GBS, consistent with earlier reports of elevated CSF BD-tau concentrations in this condition [13].

Several studies have explored the association between NfL and clinical outcomes in GBS [27, 28, 31, 32], although the precise origin, whether from the central or peripheral nervous system, remains to be established [29, 33]. Peripherin has been proposed as a novel, specific biomarker for acute peripheral axonal damage. In a study from 2023, peripherin concentrations were significantly higher in patients with GBS than in patients with chronic inflammatory demyelinating polyneuropathy (CIDP), multiple sclerosis (MS), dementia, or in healthy controls. While peripherin and NfL often increased in parallel, differences in timing and the occurrence of isolated NfL elevations in GBS suggested that peripherin more sensitively reflects peripheral nerve injury. Unlike NfL, peripherin concentrations did not appear to increase with age [34].

We found no significant differences in AINX concentrations across neurophysiological or clinical subtypes. Although median AINX concentrations were numerically higher in patients with axonal subtypes, no statistically significant differences were observed between NCS subtypes after adjustment for age. The same applied to Miller Fisher syndrome (MFS), despite our initial expectation of higher AINX concentrations due to its presumed greater extent of CNS involvement. A possible explanation is a milder clinical presentation, with fewer affected nerves and less extensive nerve damage, as none of the MFS patients had a poor outcome at 12 months. This is consistent with previous reports of a generally favorable prognosis in MFS [1].

Our cohort’s demographics align with reports from North America and Europe, suggesting that the findings are generalizable to GBS patients in similar healthcare settings [3]. However, the results should be interpreted considering several study limitations. First, the timing of CSF sampling in relation to treatment initiation varied between patients, with some samples obtained after treatment had already been started. As treatment may potentially influence biomarker concentrations, this variability could have affected the measured AINX levels. Second, the retrospective design relying on medical records meant that evaluation of functional status according to GBSDS was more difficult in some cases than in others. Likewise, fatigue at 12 months was assessed based on available documentation and not through a standardized evaluation, which may have influenced its classification. Finally, one patient in the cohort was only 3 years old, which may represent a potential source of variability given ongoing anatomical development and limitations associated with retrospective evaluation of clinical factors.

To conclude, our findings show that elevated cerebrospinal fluid AINX concentrations at diagnosis are associated with poor long-term outcome, but not with disease severity at nadir in GBS. As AINX is considered a CNS-derived biomarker, these results support previous suggestions that CNS involvement may contribute to recovery and outcome in GBS.

## Data Availability

The datasets analyzed during the current study are not publicly available due to ethical and privacy restrictions but are available from the corresponding author on reasonable request.
